# Patient-Treatment Matching: Rationale and Results

**Published:** 1994

**Authors:** Margaret E. Mattson

**Affiliations:** Margaret E. Mattson, Ph.D., is the staff collaborator for Project MATCH at the National Institute on Alcohol Abuse and Alcoholism, Bethesda, Maryland

## Abstract

Because most treatment experts now agree that no single therapy produces the best outcomes for all patients with alcohol problems, researchers are becoming interested in matching patients to certain types of treatments based on the characteristics that each patient possesses.

For many years, the desire to improve the outcome of alcoholism treatment has prompted researchers and clinicians to compare different treatments in the hope of discovering the “best treatment.” This race to find a single solution has produced a large volume of literature on the efficacy of a wide range of treatments involving social; behavioral; psychiatric; and, most recently, pharmacologic components. Although a few treatments have emerged that seem to outperform others (e.g., social skills training, motivational enhancement therapy, and the community reinforcement approach; for a more complete description of these treatments, see the article by Kadden, pp. 279–286), researchers now generally recognize that no single form of treatment is likely to be effective for all people with alcohol problems^1^ (Hester and Miller 1989; Holder et al. 1991; Allen and Kadden in press). The treatment field now seeks to obtain optimal outcomes by considering both the main effects of a treatment and the additional improvement that may result if a client is matched to the most appropriate treatment.

In alcoholism treatment research, analyses comparing the main effects of different treatments often have revealed that they have similar benefits. However, by performing analyses that examine the interactions between certain patient and treatment types, differential benefits may then become evident. That is, particular treatments work better for some patients than for others, and in some cases, treatment can be improved by “matching” subgroups of patients with the therapies most suited to their needs, based on a specific treatment’s record of success for clients of the same description.

The matching approach recognizes that alcoholism is a multifaceted disorder; thus, all treatments will not have the same effect on all people diagnosed with this condition. This perspective reframes the central question of alcoholism treatment research as follows: “What treatment, by whom, is most effective for this individual with these specific problems, and under which set of circumstances?” (Institute of Medicine [IOM] 1990, p. 143).

Although in recent years practitioners have given considerable attention to individualizing treatment, client-treatment matching is not a new concept in the alcoholism treatment field (Bowman and Jellinek 1941; Wallerstein 1956, 1957), nor is it new in psychiatry, medicine, or educational research (Snow 1991). Indeed, matching treatment to individuals is not “a unique and idiosyncratic development” but “a particular application of a general strategy in human therapeutics” (IOM 1990, p. 280). Thus, it is the way in which matching is now being evaluated, rather than the concept of matching, that is relatively new.

Considerable preliminary evidence from numerous clinical trials suggests that matching may be a promising approach to improving treatment outcomes (Mattson et al. 1994). According to the IOM:

Reason for optimism in the treatment of alcohol problems lies in the range of promising alternatives that are available, each of which may be optimal for different types of individuals (IOM 1990, p. 147).

This article reviews the role of matching in alcoholism treatment and research and describes the major features of Project MATCH, an ongoing multisite trial of patient-treatment matching (see sidebar).

**Project MATCH**

During 1989 the mounting evidence for the existence of patient-treatment matching interactions that enhanced treatment success was supported by published experimental reports and the consensus of treatment experts. This prompted the National Institute on Alcohol Abuse and Alcoholism (NIAAA) to initiate a large-scale controlled clinical trial. Named Project MATCH, the trial was designed to evaluate systematically and rigorously the hypothesis that treatment matching is effective (Project MATCH Research Group 1993). After undergoing a competitive peer review process, nine institutions were selected to receive funding as part of a cooperative agreement, an endeavor representing NI-AAA’s largest treatment research initiative to date. The first 5 years of the program concluded in the fall of 1994, when a 3-year continuation was initiated to allow further analysis of the extensive data being collected. More than 1,700 patients have been studied, representing a diverse patient pool from public and private treatment facilities throughout the United States. The methods employed in Project MATCH have been described in detail by Donovan and Mattson (1994).

***Project MATCH Hypotheses***

Hypotheses proposed in the project are intended to link a diverse range of client characteristics to differential outcomes in the various treatments. Ten main categories of patient characteristics were measured: demographic and clinical history, personality and predisposing factors, treatment history and health-service use, alcohol-related syndromes and consequences, drinking behavior and other drug use, psychosocial functioning, psychiatric disorders, neuropsychological functioning, attitudinal and motivational factors, and social support.

***Study Design***

The study sites were divided approximately evenly between two independent but parallel study arms: outpatient facilities and facilities providing aftercare to clients who already had completed an intensive inpatient or day hospitalization program. In both study arms, clients were evaluated using an extensive baseline inventory, randomized to one of the three treatments (12-step facilitation, cognitive-behavioral coping skills therapy, and motivational enhancement therapy; described below) and reevaluated at 3, 6, 9, 12, and 15 months. In addition, in the outpatient arm, patients are being followed for 39 months. These followup evaluations track drinking and other drug use, psychological and social functioning, and use of health services.

Analyses of the project’s hypotheses will demonstrate whether the patient characteristics studied are associated with differential treatment outcomes in each of the three therapies. Minorities and women are represented in the study sample in proportions equivalent to their representation in the treatment-seeking population (20 percent and 25 percent, respectively), allowing statements to be made about effects on treatment success in these sub-populations. Investigations of therapist-client interactions within treatment conditions also will be possible because of the large number of therapists (approximately 80) involved in the project.

***Treatments***

The project’s objective is to determine the way in which subgroups of alcoholics respond to three treatments, all of which are believed generally to be similar in efficacy: (1) 12-step facilitation (TSF), (2) cognitive-behavioral coping skills therapy (CB), and (3) motivational enhancement therapy (MET). The procedures and content of each therapy were analyzed in detail and have been described elsewhere (DiClemente et al. 1994; Donovan et al. 1994). Major features of the three therapies are summarized below.

***Twelve-Step Facilitation.*** The TSF program is designed to prompt the client’s active participation in traditional Alcoholics Anonymous (AA) activities that promote fellowship. A Project MATCH counselor encourages the client to attend local AA meetings regularly and use the tools offered by the fellowship: studying and practicing the 12 steps, reading and understanding AA-approved literature and slogans, using a sponsor, and receiving support from other members. Project MATCH counselors center discussions with clients around the goals of the first two steps of TSF therapy: acceptance and surrender.

***Cognitive-Behavioral Coping Skills Therapy.*** Project MATCH’s CB therapy focuses on training patients in interpersonal and self-management skills to control their drinking behavior. This therapy helps patients acquire new skills and cognitive strategies, with the goal of replacing maladaptive habits with healthy behaviors. Skills training is emphasized to enable clients to achieve and maintain sobriety. Clients learn to cope in high-risk drinking situations, practice drink-refusal skills, manage negative moods that may lead to drinking, and enhance social supports. CB therapy employs role-playing exercises during sessions as well as homework assignments to reinforce the skills learned.

***Motivational Enhancement Therapy.*** MET is based on the principles of motivational counseling. It encompasses the dual goals of building patients’ motivation for change and strengthening commitment to change. The approach aims to alter patients’ readiness to change until they reach the point at which they will take action. Early in treatment, MET provides clients with individualized feedback, from assessments made using objective questionnaires on the magnitude of and problems associated with client drinking behavior, to stimulate their motivation to change. Counselors encourage commitment to change and try to increase the clients’ awareness of their inner resources (such as their desire to improve their quality of life) as well as external supports. MET aims to present change as a force within the client, rather than something to be learned from a therapist.

All three treatments were delivered to patients in individual sessions during a 12-week period following guidelines described in standardized manuals (Kadden et al. 1993; Miller et al. 1993; Nowinski et al. 1993) by therapists trained and certified for overall counseling skills. Therapist performance was monitored routinely during the intervention phase of the trial so that any departures from protocol or lessening of treatment could be corrected.

— *Margaret E. Mattson*

ReferencesDiClementeCCCarrollKMConnorsGJKaddenRMProcess assessment in treatment matching researchJournal of Studies on AlcoholSupp 121994156162772299210.15288/jsas.1994.s12.156DonovanDMMattsonMEAlcoholism Treatment Matching Research: Methodological and Clinical ApproachesSupp 12 of Journal of Studies on Alcohol19947722998DonovanDMKaddenRDiClementeCCarrollCLongabaughRZwebenARychtarickRIssues in the selection of therapies in alcoholism treatment matchingJournal of Studies on AlcoholSupp 121994138148772299010.15288/jsas.1994.s12.138KaddenRCarrollKDonovanDCooneyNMontiPAbramsDLittMHesterRCognitive-Behavioral Coping Skills Therapy Manual: A Clinical Research Guide for Therapists Treating Individuals With Alcohol Abuse and DependenceNational Institute on Alcohol Abuse and Alcoholism Project MATCH Monograph Series3NIH Pub. No. 94–1895Rockville, MDNIAAA1993MillerWRZwebenADiClementeCCRychtarickRGMotivational Enhancement Therapy Manual: A Clinical Research Guide for Therapists Treating Individuals with Alcohol Abuse and DependenceNational Institute on Alcohol Abuse and Alcoholism Project MATCH Monograph Series2NIH Pub. No. 94–1894Rockville, MDNIAAA1993NowinskiJBakerSCarrollKTwelve Step Facilitation Therapy Manual: A Clinical Research Guide for Therapists Treating Individuals with Alcohol Abuse and DependenceNational Institute on Alcohol Abuse and Alcoholism Project MATCH Monograph Series1NIH Pub. No. 94–1893Rockville MDNIAAA1993Project MATCH Research GroupProject MATCH: Rationale and methods for a multisite clinical trial matching alcoholic patients to treatmentAlcoholism: Clinical and Experimental Research17611301145199310.1111/j.1530-0277.1993.tb05219.x8116822

## What Is Patient-Treatment Matching?

In its simplest terms, patient-treatment matching means prescribing treatment based on individual patient needs, as opposed to providing the same therapy to all patients with a common diagnosis. The ultimate goal of matching research is to develop valid, practical rules for clinicians to use in assigning patients to treatment regimens.

### The Current Practice of Assigning Treatment

Ideally, clinicians today assign patients to treatment based on criteria such as the severity of their alcoholism or the presence of co-occurring pathology or other problems (e.g., marital problems or lack of social support). In this way, clinicians can ensure that treatments address the factors that are related to the patients’ alcohol problems (IOM 1990). By doing so, clinicians apply the matching concept to their decisions. Patients also contribute by practicing “self-matching” (i.e., patients help decide on the course of treatment depending on their personal resources, beliefs about what will benefit them most, and recommendations from significant others). However, these decisions by both clinicians and clients regarding appropriate treatment may be based on personal impressions rather than on rules validated by controlled clinical research. On occasion, in clinical practice, clients may be assigned to treatment unsystematically according to the available services or to other practical factors, such as scheduling logistics.

### The Components of Matching

In research, patient-treatment matching is defined as varying the principal treatment approach that is being used from one client to another based on a precise plan. The plan includes three components: (1) systematic assignment of patients to well-defined treatments, (2) comprehensive assessment of client characteristics and needs, and (3) explicit treatment-matching guidelines or rules. These guidelines for matching should be supported theoretically and justified empirically. Outcomes should be monitored so that these guidelines can be refined further as data regarding treatment efficacy accumulate (Del Boca and Mattson in press).

## Types of Patient-Treatment Matching

### Distinction Between Outcome Predictors and Matching Factors

**Figure 1:**
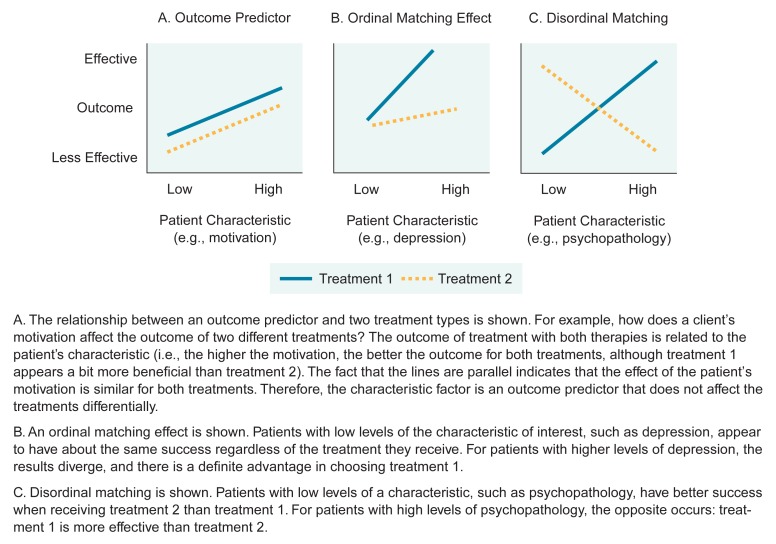
Three hypothetical examples of the relation of patient characteristics to treatment success are presented. The X axis (horizontal line) of each graph reflects the degree to which a patient has a certain characteristic. It may be a continuous variable (i.e., a patient may lie anywhere along a spectrum of characteristic levels varying from low to high), such as the degree of a patient’s motivation, or a dichotomous variable, such as the presence or absence of a family history of alcoholism (not shown). The Y axis (vertical line) is a measure of treatment outcome (e.g., the percent of days in a given period that the patient consumed alcohol). The relationship between the two lines that represent the two treatments being compared reveals information about the effects of varying levels of patient characteristics on outcome.

In developing treatment-matching guidelines, it is important to realize that some patient characteristics affect outcomes from all types of treatment in the same manner, whereas other characteristics have a distinct effect on certain types of treatments. Treatment-matching research attempts to identify those traits that have differential effects because they can be matched with treatments they affect positively. Therefore, a distinction should be made between outcome predictors, which are characteristics that have no differential effect, and matching factors, which produce different effects depending on the type of treatment used. Patients in alcoholism treatment who possess particular outcome predictor characteristics have successful outcomes regardless of the type of treatment they receive. Outcome predictors also may have negative effects on outcome; that is, patients with certain other predictor characteristics may have less favorable outcomes regardless of the treatment they receive. Matching factors, on the other hand, are patient characteristics that interact with treatment types. Patients respond to treatments differently, depending on the degree to which they possess a matching factor. This differential response provides clues to which treatments work best for particular patients and thus is the basis for treatment matching. Figure 1 and the following examples illustrate outcome predictors and two forms of matching—ordinal and disordinal (sometimes called quantitative and qualitative). The figure diagrams three types of results that may arise from studies that compare two treatment types. Both treatments are tested in groups of patients who have been shown to vary from low to high in their levels of a certain characteristic of interest.

#### Outcome Predictors

Graph A in figure 1 shows the effect of an outcome predictor, which does not produce a matching effect. The outcomes of treatment with both therapies tested improve as the levels of the outcome predictor improve. Thus, in this case, the higher the level of the predictor a patient possesses, the better is his or her outcome for both types of treatment. For example, graph A shows that increasing motivation levels among patients enhance outcome equally regardless of the type of treatment. Patient characteristics that have been identified as positive outcome predictors include the following:

- Being married
- Having a higher socioeconomic status
- Being employed
- Being motivated to change
- Having higher intellectual functioning
- Having greater ability to cope with stressors and urges to drink
- Participating in aftercare programs after an inpatient treatment program
- Having greater social stability.

(For more information on outcome predictors, see reviews by Gibbs and Flanagan 1977; Nathan and Skinstad 1987; Waisberg 1990; Westermeyer 1989; and Mattson et al. 1994.)

#### Ordinal Matching

Graph B in figure 1 illustrates one way that ordinal matching may occur. In this case, patients with high levels of a certain characteristic fare better in one type of treatment than in another, whereas patients with low levels of the characteristic appear to have approximately equal success in both types of treatment. For example, suppose that two types of treatment are given to patients whose levels of depression have been assessed and placed on a spectrum from low to high. Results show that for those patients on the lower end of the depression scale, both treatments appear to be equally effective. For those patients with higher levels of depression, however, it is clear that one treatment is more effective.

#### Disordinal Matching

This type of matching is observed when the treatment effects are reversed as patients vary from having low to high levels of the characteristic being studied (graph C). One treatment will be beneficial for patients with low levels of the characteristic and not for patients with high levels. The opposite will be true for the other type of treatment. In a published example of this effect (Cooney et al. 1991; Kadden et al. 1989), researchers found that patients with higher levels of sociopathy (i.e., being poorly socialized) and psychopathology fared better with coping skills therapy than with interactional therapy. The opposite reaction (i.e., interactional therapy produced better results than did coping skills therapy) occurred for those patients who were low in these traits.

Another illustration of disordinal matching are the results from a study in which women experienced greater success in a program that emphasized educational lectures and films than in a group therapy session format, whereas the opposite was true for men (Cronkite and Moos 1984).

## Evidence for Patient-Treatment Matching’s Effectiveness

Approximately 30 empirical studies on patient-treatment matching, spanning more than 20 years, report positive findings. Because of this lengthy time period, the methodology used in some reports lacks the sophistication expected by today’s standards, and the studies have not been replicated by other researchers. Nonetheless, the studies do support the theory of treatment matching, indicating that this approach warrants further examination. Highlights from the 30 studies are presented below, and the studies have been reviewed in detail elsewhere (Hester and Miller 1989; Mattson et al. 1994).^2^

### Classifying Patient Characteristics

The critical initial step in studying client-treatment matching is to specify the characteristics and needs of patients that may be important for selecting the best treatment. A variety of ways to classify patient variables have been described in the treatment-matching literature, including a simple four-category system consisting of demographic variables (such as gender and age), alcohol-specific characteristics (such as amount and duration of alcohol consumption and family history of alcoholism), intrapersonal characteristics (such as psychiatric status, personality characteristics, and emotional traits or states), and interpersonal functioning (including social support and social stability). Classifying patient variables into categories provides an organization for designing research and forming hypotheses for promising treatment matches.^3^ Examples of patient characteristics in these categories reported in the literature are described in more detail below, and specific treatment matches are summarized in table 1.

#### Demographic Characteristics

Several demographic factors may prove valuable when matching patients to treatments (table 1). For example, although women have been included in past treatment research studies less frequently than men, gender still is recognized as having importance. Further study may reveal that more demographic factors have matching interactions with treatments, although it is likely that, in general, demographics are oversimplifications of more complex factors. Demographics may, therefore, serve as proxies for combinations of underlying cultural, social, and psychological factors that vary along with them. In the role of proxies, demographic factors could serve either as useful summary indicators or, in some cases, mask the true effects of other variables.

#### Drinking-Related Characteristics

A wide variety of patient drinking-related characteristics have proved beneficial to treatment outcome when matched with certain treatments. Such traits include a patient’s skill in refusing to drink during role-playing, endorsement of the notion that alcoholism is a disease, a family history of alcoholism, and motivation to change drinking habits (table 1). Furthermore, it appears that alcoholics who are more dependent on alcohol and have more severe drinking problems do better in more intensive treatment (Orford et al. 1976; Edwards et al. 1977).

#### Intrapersonal Characteristics

These factors have received the most attention. Sociopathic tendencies produce differing results when matched with certain treatment types, such as coping skills and interactional therapies (figure 1, graph C; Cooney et al. 1991; Kadden et al. 1989). In addition, other patient characteristics that have shown potential for matching and that seem to indicate the need for further study include levels of psychiatric severity, cognitive intactness, conceptual level,^4^ self-image, locus of control, and field dependence^5^ (table 1).

#### Interpersonal Characteristics

Accumulating evidence from studies involving interpersonal factors suggests that the social influences that patients encounter (e.g., support or lack of support from others) have much potential for treatment matching to improve patient outcomes (Longabaugh et al. 1993) (table 1). In recent studies, the influence of social support has been defined and applied more specifically (Longabaugh et al. 1993). For example, Longabaugh and colleagues (1993) have distinguished between the more general concept of social support for one’s psychological well-being and alcohol-specific social support, which they define as “behaviors by others in the environment that stimulate and/or reinforce alcohol consumption or abstinence” (p. 465). Longabaugh and colleagues also are attempting to explain the role of social investment, or a person’s dependence on other people for differential reinforcement or rewards, as an additional factor in how a person’s drinking is affected by social influences. Conceptual refinements such as these are important for understanding more precisely how others in society influence an individual’s ability to control drinking.

## Variations on the Matching Theme

The previous discussion has emphasized matching psychosocial treatments to particular patient characteristics, and most research to date has focused on this type of matching. However, other approaches exist for matching treatments and clients to achieve optimal effects. Some possibilities are discussed below.

### Pharmacologic Interventions

The development of systems for matching patient characteristics with pharmacologic agents alone or in combination with psychosocial therapies has not been explored often but is receiving increasing attention. In one study, patients with a family history of alcoholism had better success with treatment while taking the antidepressant fluoxetine, whereas those patients without a family history did better using another agent, Ca-acetyl-homotaurinate (Gerra et al. 1992) (see the article by Anton, pp. 265–271). In another study, the anxiolytic agent buspirone was shown to be more effective for treating anxious alcoholics than alcoholics with low levels of anxiety (Kranzler et al. 1994).

### Self-Matching

Two additional treatment matching approaches include (1) self-matching, or client-directed treatment selection, and (2) the “cafeteria approach” (Ewing 1977), which recommends offering clients a menu of treatment options as well as providing varying levels of guidance from the clinician. It is felt that promoting completely self-directed choices among patients is unwise, because clients may feel confused when faced with many options or may simply be unable to make choices that are in their own best interests (Lindstrom 1992). Generally, however, an appropriate degree of patient involvement in treatment planning may enhance the patient’s commitment to the program (Miller 1989).

### Therapist-Patient Interactions

Because evidence suggests that certain characteristics that therapists possess may influence treatment outcome (Luborsky et al. 1985; Miller et al. 1980), it is possible that a therapist’s qualities, if paired with the most compatible kind of client or treatment, may lead to greater treatment success. Matches may be based on situations in which the therapist’s characteristics (listed below) interact so strongly with the patient’s characteristics that these effects (e.g., the therapist’s ability to motivate the patient) may benefit the patient as much as or more than does the formal intervention the therapist is using. For example, Miller and colleagues (1993) found that patients who viewed alcoholism as a bad habit had better outcomes with therapists who were empathetic rather than confrontational, although no difference in outcomes was observed for those patients who viewed alcoholism as a disease. Also, situations in which some counselors are particularly effective in certain types of therapies, called therapist-treatment interactions, are possible.

Therapist characteristics that have been reported to produce successful outcomes in psychotherapy include personal style, genuineness, respect for clients, and concreteness; personal adjustment and ability to help others; disclosure of personal experiences that are therapeutically relevant to the patients’ situations; gender; empathy with clients, positive regard for clients, and unconditionality; and whether the therapists themselves are recovering problem drinkers.

### Matching As a Dynamic Process

Matching may be a dynamic process that evolves as the client’s beliefs, behavior, and environment change over time. As recovery proceeds, the patient’s changing needs may warrant a variation in therapy, or even a new treatment approach, one or more times during the rehabilitation process. Assuming that the recovery process involves several stages, as some research hypothesizes (Finney and Moos 1986; Prochaska et al. 1992; DiClemente and Hughes 1990), the dynamic treatment-matching perspective could be applied in several ways. Certain interventions may be more useful at particular stages of recovery than others; with appropriate guidelines, practitioners could decide when these transition periods occur and what modifications in the intervention strategy are required.

For example, Prochaska and colleagues (1992) and DiClemente and Hughes (1990) suggest that five stages are involved in the modification of addictive behavior: precontemplation, contemplation, preparation, action, and maintenance. Patients in the earlier stages may require interventions that assist them in increasing their motivation to complete treatment, as well as insight and understanding of their drinking problems. In contrast, those patients in the action stage of the process may need help in acquiring new behaviors, such as learning drink refusal skills in social situations.

## Methodologic Requirements

The field of treatment matching is gathering momentum as studies accumulate and as methodologic advances are practiced more commonly. The following is a list of strategies that could contribute to stronger research designs and hence more conclusive results:

- The selection and measurement of client characteristics for study must be considered carefully, with theory or some *a priori* rationale guiding the process.
- The treatments compared must be specified and distinct so that content overlap among the types of treatment does not confound the search for matching interactions. Unfortunately, the capability to describe treatment programs is not as advanced as the capability to describe client characteristics, and models and surveys of the content of active treatment programs are needed (IOM 1990).
- When a successful treatment match is found, the mechanism of action underlying the interaction between the client characteristic and the treatment principle must be understood.
- Treatment delivery needs to be true to protocol and consistent over the course of the study and across multiple therapists and sites.
- Outcome measures and criteria for client improvement need to be appropriate to the treatment goal and assessed in a standardized way to make comparison across studies possible.
- Treatment-matching studies require large sample sizes that may demand use of multiple and geographically distant clinical sites, thereby presenting a variety of logistical, management, and quality-assurance challenges.

## Implementation Challenges

The ultimate goal of treatment-matching research is to provide clinicians with tested and practical matching algorithms, or “rules,” that will enable clinicians to make more informed treatment-planning decisions for their patients. Hopefully, better decisions will enhance outcomes, reducing the personal, social, financial, and medical costs of alcohol misuse. But what caveats to this hope can be anticipated? No doubt some patients will not get better, no matter what treatment they receive, and other patients will get better despite what treatment they receive. In many cases, however, patients’ improvements will be modest in magnitude, no matter what treatment they receive.

Apart from these limitations (which are by no means unique to alcoholism treatment) lies the practical problem of translating research findings that studies such as Project MATCH may deliver into clinical practice (see sidebar). This is not an automatic process, as evidenced by the observation that those treatment strategies that research generally has shown to be more effective are not necessarily the ones most commonly found in clinical practice (Miller 1987).

Both the researcher and the clinician have important roles in facilitating the transfer of new treatment techniques. They must share information on new developments in treatment matching as well as other treatment advances. Researchers can greatly enhance this process by disseminating new results through publications and conferences that are accessible to treatment providers in many fields and in a format understandable to the nonresearcher. Practitioners may benefit from new findings, not only by reading literature and attending continuing education activities but also by keeping an open mind to the need to change established patterns of providing treatment.

Venues that promote dialogue between providers and researchers are needed to ensure that the requirements and constraints of clinical practitioners are communicated to those responsible for planning research. Administrators of treatment facilities may play an essential role by providing incentives for therapists to integrate validated treatment improvements into their practices. Such incentives could include institutional support of staff education and a willingness to examine existing delivery systems for barriers to implementation of improved treatment techniques. Insurers already exert a fundamental control over how treatment is conducted by relying on increasingly strict standards of care as a reimbursement criterion.

## Conclusion

Through rigorous validation of matching hypotheses and production of practical matching strategies, treatment-matching research should contribute new clinical strategies to control alcohol abuse and dependence. It is hoped that this promising tool will contribute to improvements in both the physical health and the quality of life of people striving to attain and maintain command over their drinking behavior.

## Figures and Tables

**Table 1:** Patient-Treatment Matching Findings From the Literature

Reference	Patient Variable	Treatments	Match^1^
	**Demographics**		

Cronkite and Moos (1984)	Gender	In-depth therapy (IT—group therapy) vs. Education sessions (ES—films and lectures)	Males: IT>ES Females: ES>IT
Babor and Grant (1992)	Gender	Simple advice (SA—20-minute health interview plus 5 minutes of advice about abstinence or sensible drinking) vs. Brief counseling (BC—20-minute health interview plus 15 minutes of counseling, a self-help manual, and followup sessions) vs. Control (C—20-minute health interview)	Males: SA=BC>C Females: SA=BC=C
Scott and Anderson (1990)	Gender	10-minute Physician advice (PA—health survey given plus a 10-minute feedback-and-advice session about drinking less and a self-help booklet) vs. Usual care (UC—health survey given. No advice unless requested)	Males: PA>UC Females: PA=UC

	**Drinking-Related Characteristics**		

Annis and Davis (1989)	“Differentiated” drinker (able to recognize situations prompting drinking)	Relapse prevention therapy (RP—focuses on identifying patients’ high-risk drinking situations and how to handle them) vs. Traditional counseling (TC)	Differentiated: RP>TC Undifferentiated: RP=TC
Miller et al. (1993)	Clients view of alcoholism (bad habit vs. disease)	Confrontive therapist (CT—therapists confront patients’ minimization of problem with evidence of alcohol problems) vs. Nonconfrontive therapist (NCT—therapists respond in an empathetic manner and make no direct attempts to prove points about alcohol problems)	Bad habit view: NCT>CT Disease view: NCT=CT
Gerra et al. (1992)	Family history with respect to alcoholism	Fluoxetine (F) vs. Ca-acetyl-homotaurinate (H) (each medication acts through a different mechanism to reduce drinking) vs. Placebo (C)	+Family hx: F>H=C −Family hx: H>F=C

	**Intrapersonal Characteristics**		

Kadden et al. (1989); Cooney et al. (1991)	Sociopathy (being aggressively antisocial)	Interactional skills (IS—group therapy that explores participants’ interpersonal relationships) vs. Coping skills (CS—highly structured group therapy focusing on learning problem-solving and interpersonal skills)	Hi socio: CS>IS Lo socio: IS>CS
Hartman et al. (1988)	Locus of control (internal vs. external)	Brief nondirective treatment (BND—three individual counseling sessions given at 1-month intervals. Focus on identifying treatment goals and giving pragmatic advice) vs. Intensive structured treatment (INS—eight group sessions over 4 weeks with followup. Focus on coping skills training^2^)	Internal: BND>INS External: INS>BND
Annis and Chan (1983)	Self-image (SI)	Intensive group confrontive (IGC—centered on highly confrontational, 2-hour group therapy sessions) vs. Standard institutional program (SIP)	+SI: IGC>SIP −SI: SIP>IGC
Welte et al. (1981)	Social stability (SS)	Longer inpatient (L—more than 60 days) vs. Shorter inpatient (S—less than 60 days)	Lo SS: L>S Hi SS: L=S
Longabaugh et al. (1993)	Social investment (SI) (person’s dependence on others for differential reinforcement)	Relationship enhancement cognitive behavioral (RE—involves significant others in learning how a relationship can reinforce abstinence and deal with slips) vs. Individual focus cognitive behavioral^2^ (IF—teaching problem-solving techniques and how to deal with slips)	Lo SI: IF>RE Hi SI: RE=IF

1 A shorthand notation has been devised to summarize patient-treatment matching results. It presents outcomes for the treatments being compared as they relate to the level of the client characteristic of interest using standard equality and inequality symbols to denote better, worse, or equal outcomes. For the Annis and Davis study, for example, the notation means that alcoholics identified as “differentiated” (i.e., they are able to identify stimuli to drink) had better outcomes from the relapse prevention therapy than from traditional counseling. Conversely, among those classified as “undifferentiated,” no difference was seen in treatment effects: differentiated: RP>TC undifferentiated: RP=TC

2 For a more detailed discussion of this and other cognitive-behavioral techniques, see the article by Kadden, p. 279–286.

## Footnotes

1 In this article, the term “alcohol problems” refers to any problems a person experiences that are related to the consumption of alcohol. The terms “alcoholism” and “alcohol abuse” refer to the definitions of substance abuse and dependence described in the American Psychiatric Association’s *Diagnostic and Statistical Manual of Mental Disorders, Fourth Edition*.

2 Further informative background is found in the work of Longabaugh and colleagues, who have described in detail the nature and clinical implications of the two main types of matching defined above as well as the many possible variants (Longabaugh et al. 1994). Lindstrom has reviewed the evidence supporting matching in a recently published book (Lindstrom 1992). Finally, several conceptual articles have discussed the underlying rationale and the research design requirements of matching (Gottheil 1981; Finney and Moos 1986; Miller 1989).

3 Except in a few instances, the broad diversity of interventions studied and the lack of sufficient systematic replication make widespread translation to clinical practice premature. Furthermore, uniformity of research methodology is still at an early stage, making comparisons across studies difficult in many cases (Mattson and Allen 1991).

4 Conceptual level is a personality orientation that defines higher conceptual levels as being cognitively complex, empathetic, and tolerant of ambiguity. Lower levels are considered cognitively simple, egocentric, and more dependent upon rules.

5 High field dependence is a perceptual trait that has been suggested to reflect a personality type characterized by, among other things, a less-developed sense of self-identity.
